# Azacitidine prolongs overall survival and reduces infections and hospitalizations in patients with WHO-defined acute myeloid leukaemia compared with conventional care regimens: an update

**DOI:** 10.3332/ecancer.2008.121

**Published:** 2008-12-10

**Authors:** P Fenaux, GJ Mufti, E Hellström-Lindberg, V Santini, N Gattermann, G Sanz, AF List, SD Gore, JF Seymour, J Backstrom, L Zimmerman, D McKenzie, CL Beach, LB Silverman

**Affiliations:** 1 Hôpital Avicenne, Université Paris 13, Bobigny, France; 2 Department of Haematological Medicine, Kings College London, London, UK; 3 Karolinska University Hospital, Stockholm, Sweden; 4 Hematology, Azienda Ospedaliera Careggi, Firenze, Italy; 5 Heinrich-Heine-University, Düsseldorf, Germany; 6 Department of Hematology, Hospital Universitario La Fe, Valencia, Spain; 7 H Lee Moffitt Cancer Center and Research Institute, Tampa, FL, USA; 8 The Sidney Kimmel Comprehensive Cancer Center, Johns Hopkins, Baltimore, MD, USA; 9 Department of Haematology, Peter MacCallum Cancer Institute, Victoria, Australia; 10 Celgene, Overland Park, KS, USA; 11 Pediatric Oncology, Dana-Farber Cancer Institute, Boston, MA, USA

## Abstract

Azacitidine (AZA), as demonstrated in the phase III trial (AZA-001), is the first MDS treatment to significantly prolong overall survival (OS) in higher risk MDS pts ((2007) *Blood* **110** 817). Approximately, one-third of the patients (pts) enrolled in AZA-001 were FAB RAEB-T (≥20–30% blasts) and now meet the WHO criteria for acute myeloid leukaemia (AML) ((1999) *Blood* **17** 3835). Considering the poor prognosis (median survival <1 year) and the poor response to chemotherapy in these pts, this sub-group analysis evaluated the effects of AZA versus conventional care regimens (CCR) on OS and on response rates in pts with WHO AML.

## Methods

The AZA-001 trial enrolled higher risk MDS pts (FAB: RAEB, RAEB-T, CMML and IPSS: Int-2 or High). Prior to randomization, site investigators pre-selected (based on age, performance status and co-morbidities) one of three CCR: best supportive care only (BSC); low-dose ara-C (LDAC) or intensive chemotherapy (IC). Pts were subsequently randomized 1:1 to AZA (75 mg/m^2^/d SC × 7d q 28d) or CCR; pts randomized to CCR received their investigator pre-selected treatment. Karyotypes were reclassified using AML standards: favourable (inv 16, t(8;21)), unfavourable (−7/7q- or complex) and intermediate (all others including normal). OS was assessed by Kaplan-Meier (KM) methods and Cox proportional hazards model, and IWG AML criteria (2003 *J Clin Oncol* 214642-9) were used to assess morphologic complete remissions (CR). Efficacy analyses included all WHO AML pts randomized. All pts were followed until death or study closure.

## Results

Of 358 enrolled pts, 113 met the definition for WHO AML (median: 23% blasts) of whom 86% were considered unfit for IC and were pre-selected by investigators to receive a low-intensity regimen (BSC or LDAC). Fifty-five of the 113 pts were randomized to AZA and 58 pts to CCR. AZA and CCR groups had comparable baseline demographic and clinical characteristics. Of the 58 pts randomized to CCR, five withdrew without receiving treatment, and 53 were treated with their investigator pre-selected treatment as follows: IC (19%; 10/53), LDAC (34%; 18/53) and BSC (47%; 25/53). Of the 55 pts randomized to AZA, two withdrew without receiving treatment. Median age was 70 years; 24% had an unfavourable karyotype, 72% had an intermediate karyotype (including 46% normal); no pts had a favourable karyotype. Median follow-up for OS was 20.1 months. Median (min–max) number of treatment cycles was eight (1–39) for AZA, 2.5 (1–3) for IC; 5.5 (1–14) for LDAC; and six months (2–19) for BSC. KM median OS was 24.5 versus 16.0 months, respectively, in the AZA and CCR groups, hazard ratio (HR)=0.47, 95% CI, 0.28 to 0.79, p=0.004.

**Figure 1:**
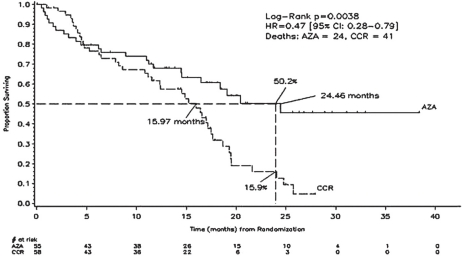
The overall survival rates in the azacitidine and conventional care regimen groups over a 40 month period.

The OS rates at two years were 50% and 16% (Figure 1), respectively, in the AZA and CCR groups, p=0.0007. There was no statistical difference in the morphologic CR rate between the AZA (18%, 10/55) and CCR groups (16%, 9/58; p=0.80). OS results in cytogenetic intermediate pts showed a significant HR, favouring the AZA group (*N*=38) over CCR (*N*=43, HR= 0.47 [95% CI: 0.24, 0.91], p=0.024) but not in pts with unfavourable cytogenetics: AZA (*N*=14) versus CCR (*N*=13, HR=0.66 (95% CI: 0.26, 1.68), p=0.381); however, pt numbers were low. WHO AML pt outcome measures showed significant benefits with AZA: fewer infections requiring IV antibiotics per pt-year in the AZA group (0.58) versus CCR (1.14, HR=0.51 [95% CI 0.29, 0.78], p=0.003) and reduced rates of hospitalization in the AZA group (3.4 per pt-year) versus CCR (4.3 per pt-year, HR=0.79 [95% CI 0.62, 1.00], p=0.028). AZA was generally well tolerated.

## Conclusion

Azacitidine significantly prolongs OS with significant improvements in important pt outcomes in elderly WHO AML pts with low-marrow blast counts, who currently have limited therapeutic options. Trials are ongoing to confirm the effect of AZA in elderly AML pts with more proliferative disease.

